# Global database of paleocurrent trends through the Phanerozoic and Precambrian

**DOI:** 10.1038/sdata.2015.25

**Published:** 2015-06-09

**Authors:** Leonard Brand, Mingmin Wang, Arthur Chadwick

**Affiliations:** 1 Earth and Biological Sciences, Loma Linda University, Loma Linda, CA 92354, USA; 2 516 E. Glenn Ave., Apt 108, Auburn, AL 36830, USA; 3 Department of Geology, Southwestern Adventist University, Keene, TX 76028, USA

**Keywords:** Sedimentology, Tectonics, Geology

## Abstract

Paleocurrents are sedimentological features contained in all sedimentary deposits, enabling the direction of movement of the sediment and the containing fluid at the time of deposition to be determined. This database contains paleocurrent directions and other relevant associated data from published sources and theses and dissertations for the entire Phanerozoic and Precambrian for all continents. Such information may be of general interest to sedimentologists and will be of specific interest in sedimentary basin analysis, and to petroleum geologists and mineralogists seeking source areas. Paleocurrents may also be useful in plate reconstructions and in testing the timing of global tectonic events.

## Background & Summary

The history of interest in paleocurrents goes back to the middle 1800s, but serious work on them began in the 1960’s. Potter and Pettijohn’s classic work^1^, published first in 1963, framed the rapid expansion of interest in paleocurrents seen in the geological literature, as sedimentologists and petroleum geologists recognized the usefulness of paleocurrents for establishing provenance and defining sedimentary basins. Paleocurrents also played a significant role in establishing plate tectonics as a viable model^2^.

This project began as an outgrowth of an assignment in a graduate sedimentology class using Potter and Pettijohn’s book. Students were challenged to recover paleocurrent data from the published literature and to use that data to test models of basinal sedimentation. The results encouraged us to continue the study. In particular, this pursuit was stimulated by the textbook authors’ challenge: ‘An aspect of paleocurrents, still far from being exploited, is compilation of more region-wide paleocurrent and facies maps... of regions exceeding a thousand or more kilometres in length and width^3^’. To accomplish this objective would require the accumulation of data on a heretofore unavailable scope. This we sought to accomplish. This phase of the work continued until the mid-1980’s when one of us (A.C.) took a new position. Subsequently the class was no longer offered and from that point the work was continued sporadically as time allowed.

The study of paleocurrent structures has long provided useful information to sedimentologists and stratigraphers. Paleocurrents have contributed to our understanding of flow directions of paleorivers^4^, longshore currents^5^, mass transport flows^6^ and paleowinds^7^. They have enabled us to decipher original paleoslopes^8^, and in unimodal systems, have given clues about provenance of sediments^9^. They continue to be useful for interpreting paleoenvironments^10^ and depositional processes^11^. Paleocurrents, along with other kinds of data have contributed to defining basinal geometry^12^ and basinal processes^13^. They have directed in the exploration for placer deposits^14^ and petroleum reservoirs^15,16^. For these and other reasons, a database of paleocurrent data can be a valuable resource for geologists working in a variety of disciplines.

Over time, we have continued to extract paleocurrent data from the literature. We developed a computer program to handle the data, and display it graphically. As our work continued, by the mid 1990s we had exhausted the readily available published records, but we found a new resource in geological theses and dissertations which often contained abundant data that were not published elsewhere. We changed our focus to this new source and began to acquire these data from universities around the world. Geology departments at nearly every major university in North America, in Australia, in England, in South America, and in Spain were visited and theses and dissertations were pulled and relevant parts were copied and later analysed for data. This work was carried out whenever it could be scheduled along with our other research projects. At present there are just over 1,000 references from bachelors, masters and doctoral theses in the database.

In the late 1990s and beyond, published papers with paleocurrent data began to be available on the internet. As a result, we were able to add extensive coverage of areas such as mainland China that have not been readily accessible until recently. A graduate student, Mingmin Wang joined our project in 2011 to help us read and interpret these papers. She continued to work with us on other areas as well. We have also begun to backfill from the published literature for the period extending back to the 1990s. It is our intent that this database will be supplemented with additional datasets over time. This is an ongoing work, but we felt that the database itself would be useful to other investigators in its present and growing form.

**Figure 1:**
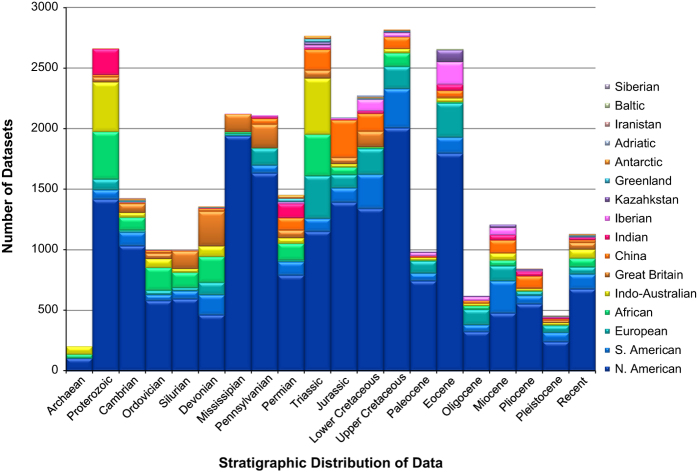
Plot of the distribution of paleocurrent datasets by stratigraphic interval (abscissa) and geographic area (stacked bars). A total of 30,136 individual datasets are represented in the diagram.

Data were acquired principally from North America during the initial phases of the study. With time, the study expanded into other areas of the world as it became apparent that paleocurrents patterns existed on the larger scales that Potter and Pettijohn had anticipated. The geographic and stratigraphic distributions of the datasets in the database are depicted in Fig. 1.

The great abundance of paleocurrent datasets (Fig. 1) in the Proterozoic is a reflection of the greater abundance of clastic rocks found there compared with the overlying Paleozoic rocks. The decreased level of paleocurrent records for the post-Cambrian Palaeozoic likely results from the abundance of limestone at the expense of clastic rocks found there. Geologists rarely report paleocurrents from carbonate rocks, even though they are often derivable from those rocks^17^. The diminished coverage in epochs of the Cenozoic is in part (Paleocene, Oligocene), a reflection of the relative scarcity of these rocks in North America.

**Figure 2:**
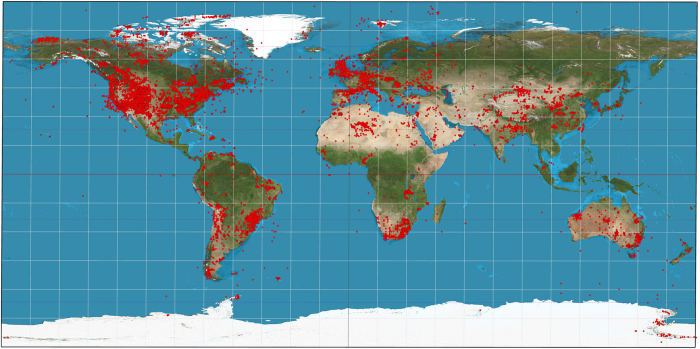
Paleocurrent datasets plotted on equirectangular projection image. One red dot represents a single dataset in the database. Background image modified from Strebe (Creative Commons Attribution-Share Alike 3.0 Unported license).

As presented here, the database contains 30,135 datasets representing over one million discrete paleocurrent determinations on outcrop (or in a few cases in well cores). The global geographic distribution of the data is represented in Fig. 2.

## Methods

Initially, the research involved examining published reports in geological journals. The following data were acquired from each publication: full citation (authors, title, journal, year, volume and pages), the stratigraphic position of the beds and the name of the formation, the latitude and longitude of each recorded dataset, the direction the current was moving, and the number of actual measurements. Additional data collected for each record included the area in square kilometres from which the dataset was obtained, the degrees of dispersion for the dataset, the continent (or plate) from which the data were derived, the environment of deposition inferred by the author, the type of paleocurrent indicators used, and the lithology. Each paper contained from one to many entries in the database, depending upon how many individually reported paleocurrent datasets were included. For example, if a paper included a map displaying five separate rose diagrams summarizing the outcrop measurements in five areas, then five entries were made in the database, one for each rose diagram. Thus, as used here, **dataset** refers to a single depiction of paleocurrent data in a paper, including the results of one or more outcrop determinations of paleocurrent direction, and would be represented by a single entry in the database.

Because there is no standard method of reporting the results of paleocurrent measurements, the data are inherently variable. For example, one paper may report one paleocurrent measurement for a 500 square km area. Another paper may report 500 measurements from one square km. Both are providing data, and thus both would be included in the database, and the additional information on the area and the number of measurements and the dispersion of the data can be used to try to compensate for the vagaries. There is also variability related to the method in which the data were presented. Some papers displayed every measurement as a discrete data point. Other papers may have the paleocurrent data grouped into rose diagrams or simply represented by a single directional sign, generally an arrow. In some cases the paleocurrent datasets were expressed in writing without any graphic. All of these would be included in the database, but only papers where the paleocurrent data were derived by actual field measurements were considered for inclusion. As indicated above, one of our goals in assembling these data was to look for trends on large scale trends, and it is our intent to do this. However, attempts to combine data from such inhomogeneous sources remain problematic. Rao and Sengupta^18^ have developed statistical methods for comparing data from multiple sources that appear promising for dealing with inhomogeneity of sources, but these methods have not yet been applied to the data in this database.

Where data were displayed as rose diagrams, a single direction was recorded for each diagram. This would be the vector mean direction if given. In cases where no vector mean was recorded, for rose diagrams, an estimate of the vector mean direction was computed by balancing the petal areas about a line, and deriving the direction using a protractor. In other cases, where an arrow was used to represent the data, the protractor was employed to obtain a direction from the arrow. Information about the data dispersion was obtained by estimating the number of degrees covered by the data, ignoring outliers. Thus, in the case of rose diagrams, the original rose could be approximately reconstituted if desired. Truly bidirectional data (as opposed to linear data with no defined sense) were represented by two datasets in the same geographic position.

Every effort was made to capture the data as presented in the papers, and to exclude any speculations that were based upon criteria other than physical, measurable parameters. Papers in which the author inferred source areas based on models or other criteria which did not include empirical support were not included. No record was made as to whether the author(s) applied palinspastic corrections to the data, or whether those corrections were warranted. In most cases, such corrections were not required, because the data were obtained from sediments which were uncomplicated tectonically. However in those cases where such corrections were warranted (and often were applied), it would be the responsibility of the user of the database to determine the suitability of the data.

## Data Records

The paleocurrent dataset is provided in xlsx format in Dryad (Data Citation 1). It contains the following fields for each record (Table 1).[Table t2][Table t3][Table t4][Table t5][Table t6]

## Technical Validation

Data were accumulated and accessioned over a period of years by a large number of individuals. Because some judgments were necessarily made, and geographic coordinates were assigned to the data, there are many places in the dataset where quality control was applied. The data were tested for geographic accuracy by checking the positional data for each continent with the boundaries of the continent in question. They were also checked visually by uploading the data to Google earth and evaluating the point distributions. The data appear to be accurate and valid geographically from these tests. Directional assignments and other data associated with each dataset were checked by at least two individuals.

## Usage Notes

The data as presented are suitable for analysis of paleocurrent trends over much of the world, and should prove useful for basin analysis and for tracing other local and regional sedimentary patterns. A program written in CPP allows the data to be graphically represented on a map of the world. The program and instructions for its use are available from the authors.

## Additional Information

**How to cite this article:** Brand, L. *et al.* Global database of paleocurrent trends through the Phanerozoic and Precambrian. *Sci. Data* 2:150025 doi: 10.1038/sdata.2015.25 (2015).

## Supplementary Material



## Figures and Tables

**Table 1:** Descriptions of Elements in Database

**Column**	**Element**	**Type**	**Description**
1	Continental Area	text	Geographic area containing dataset
2	Stratigraphic Level	text	Stratigraphic position of dataset
3	Record Number	integer	A unique descriptor for each data source
4	Author	text	List of source authors*
5	Title	text	Publication title*
6	Date	integer	Year of publication*
7	Journal	text	Source of publication*
8	Volume	integer	Volume number of publication*
9	Page	integer	Starting page*
10	to Page	integer	Ending page*
11	# records	integer	Number of datasets from this source*
12	Period	integer	Stratigraphic position of sediments in this dataset (Table 2)
13	Longitude	float	Longitude in decimal degrees
14	Latitude	float	Latitude in decimal degrees
15	Direction	integer	Direction current was moving toward in degrees
16	# Data	integer	Number of measurements in this dataset
17	Area sq km	integer	Number of sq km over which data were gathered
18	Dispersion	integer	Relative tightness of data
19	Continent	integer	Plate carrying data in this dataset (Table 3)
20	Environment	integer	Depositional environment assigned by author (Table 4 (available online only))
21	Indicator	integer	Type of measurements used to determine direction (Table 5 (available online only))
22	Lithology	integer	Type of sediment yielding paleocurrent data (Table 6 (available online only))
23	Formation	text	Sedimentological unit yielding paleocurrent data†
Continental Area—The geographic position of the dataset. The primary sort of the database.			
Stratigraphic Level—The stratigraphic position of the dataset. This is the secondary sort of the database.			
Reference number—This is a chronologically assigned number that identifies each data source. Where multiple datasets were obtained from a given reference, subsequent datasets are identified to the same reference number. The following seven categories, that identify the publication, are included only once in the database for each reference source, even if several datasets are obtained from the reference.			
Author—The names of the author(s) of the cited work.			
Title—The title of the paper from which the data were derived.			
Year—The year of publication.			
Source—The journal title, or thesis institution, or other indicator of the source of the report.			
Volume—The volume of the periodical.			
Page—starting page.			
To page—ending page.			
# records—tally of number of datasets obtained from this source.			
Period—Stratigraphic position is given by a five digit numeric code used to allow approximate placing of sedimentary units in a proper stratigraphic context. The values are unique for a given formation. Thus data from diverse sources derived from a given formation will plot to a single number. The scheme is as follows (see Table 2).			
When a new formation is encountered that is not registered in the database, its name is recorded in one data entry in the **Formation** column of the database (see below). From information given in the paper, a five digit number corresponding to the **Period** is obtained, in which the first digit is the Era, the second is the Period (or Stage of the Cenozoic), the third digit is the Stage, and the last two are assigned so as to approximate the position of this unit relative to other units of that particular stage. The number 21001 would be Paleozoic (2), Cambrian (1) entire Cambrian represented (0), first formation (01). The number 44559 would be Cenozoic (4), Miocene (4), middle (5), formation 59. The number 11933 would be Precambrian (1), Proterozoic (1), bridging over to Cambrian (9), formation 33. The last two digits make certain that the name of the formation can be reassociated with the data within our Paleocurrent program (see under Usage Notes below)			

*Given once for each data source

^†^ Given once for each unique unit.

**Table 2 t2:** Stratigraphic assignment

**1st Digit**		**2nd Digit**		**3rd Digit**	**4th and 5th Digits**
Cenozoic	4	Holocene	7	Upper=6, Mid=4, Lower=1 with 9 used for formations including next epoch up (i.e., 04903=Mio-Plio) and 0 reserved for entire interval (i.e., 04015=all Mio)	Assigned to specific formation names, or descriptive terms when formation names do not exist or are not applicable (as in Recent)
		Pleistocene	6		
		Pliocene	5		
		Miocene	4		
		Oligocene	3		
		Eocene	2		
		Paleocene	1		
Mesozoic	3	Cret-Paleoc	5	In Mesozoic and Paleozoic epochs are assigned progressively greater numbers, still with 0 and 9 as above	
		U. Cretaceous	4		
		L. Cretaceous	3		
		Jurassicc	2		
		Triassic	1		
Paleozoic	2	Permian	7		
		Pennsylvanian	6		
		Mississippian	5		
		Devonian	4		
		Silurian	3		
		Ordovician	2		
		Cambrian	1		
Precambrian	1	Proterozoic	1	In Precambrian, eras are assigned progressively greater numbers as above	
		Archaean	0		
					
Longitude—in decimal degrees.					
Latitude—in decimal degrees.					
Direction—the direction the depositing flow was moving towards in degrees. This is a vector mean direction where provided in the paper. Otherwise it is an estimate of the vector mean direction generated as indicated in the Methods section.					
#data—how many outcrop measurements are pooled in this dataset.					
Area sq km—the approximate surface area of the outcrop from which measurements were derived.					
Dispersion—Estimation of the degrees in a circle over which the data are dispersed.					
Continent—The data are at present distributed across 23 plates that can be considered separately. The defined regions, subject to future modification as needed, are presented in Table 3.					

**Table 3 t3:** Division of globe into plates and subplates

**Number**	**Plate**	**Notes**
1	African	(all)
2	Indo-Australian	(all)
3	North American	(excluding Appalachia)
4	South American	(all)
5	European	(excluding Baltic region)
6	Antarctic	(all)
7	Indian	
8	Great Britain	
9	China	
10	Siberian	(Russia above China)
11	Kazakhstan	(west of India)
12	Baltic	(eastern Europe)
13	Adriatic	(southern Europe)
14	Iberian	(Spain)
15	Anatolia	(Turkey)
16	Iranian	
17	Volgastan	(betw Black and Caspian)
18	Baluchistan	(west of India)
19	Baghdadistan	(west of Iran)
20	Madagascar	
21	Appalachian	(Florida)
22	Greenland	
23	Newfoundland	
Environment—The depositional environment assigned by the author of the publication to the sediments from which the paleocurrent dataset was derived. Papers where the designation of the author(s) was not clear or was equivocal were generally placed in the alluvial category. Environmental designations used in this database are defined in Table 4 (available online only).		

**Table 4 t4:** Environment of deposition for relevant formation or unit

**Number**	**Environment**
1	marine general
2	marine shallow
3	marine deep
4	lacustrine
5	fluvial deltaic
6	fluvial
7	alluvial
8	subaerial (eolian)
Paleocurrent indicator—the bedding feature used to acquire the paleocurrent data. The definitions used in the database for this feature are given in Table 5.	

**Table 5 t5:** Type of paleocurrent indicator used to derive flow direction from the unit in question

**Number**	**Bedding feature**
1	crossbedding
2	ripple marks
3	paleocurrent indicator not specified
4	sole marks
5	fossil orientation
6	wind direction
7	current direction
8	turbidity currents
9	topography
10	miscellaneous
11	slumps and folds
12	flute/grooves
13	imbrication
14	channel axes
15	parting lineations
16	model
17	provenance
18	sed thickening
19	electric log/dip log
20	grain orientation
Lithology—What was the rock type dominant in the deposit as indicated by the author of the report. The lithologies recorded in this database are indicated in Table 6.	

**Table 6 t6:** Lithology of the unit from which the paleocurrent data were derived

**Number**	**Lithology**
1	sandstone
2	shale
3	siltstone or turbidites
4	conglomerate
5	limestone
6	carbonate sand
7	volcanic or glacial
Formation—This column contains one instance of the name of each formation for which there is at least one paleocurrent dataset in the database. It establishes the linkage between the formation name and the specific period designation representing that formation or unit.	
